# Chimeric Antigen Receptor T Cells Targeting Cell Surface GRP78 to Eradicate Acute Myeloid Leukemia

**DOI:** 10.3389/fcell.2022.928140

**Published:** 2022-08-04

**Authors:** Wei Yu, Hang Zhang, Yuncang Yuan, Jie Tang, Xinchuan Chen, Ting Liu, Xudong Zhao

**Affiliations:** ^1^ Laboratory of Animal Tumor Models, Frontiers Science Center for Disease-Related Molecular Network, West China Hospital, Sichuan University, Chengdu, China; ^2^ Department of Hematology, Institute of Hematology, West China Hospital of Sichuan University, Chengdu, China

**Keywords:** GRP78, CAR T cell, acute myeloid leukemia (AML), hematopoietic stem cells (HSCS), cell surface

## Abstract

Acute myeloid leukemia (AML) is a serious, life-threatening hematological malignancy. The treatment outcome of relapsed or refractory AML patients remains dismal, and new treatment options are needed. Chimeric antigen receptor (CAR) T cells have been successful in improving the prognosis for B-lineage acute lymphoblastic leukemia and lymphoma by targeting CD19. However, CAR T-cell therapy for AML is still elusive, owing to the lack of a tumor-specific cell surface antigen and spare hematopoietic stem cells (HSCs). This study generated a novel CAR construction that targets the cell surface protein glucose-regulated protein 78 (GRP78) (csGRP78). We confirmed that GRP78-CAR T cells demonstrate an anti-tumor effect against human AML cells *in vitro*. In xenograft models, GRP78-CAR T cells effectively eliminate AML cells and protect mice against systemic leukemia, in the meanwhile, prolonging survival. In addition, GRP78-CAR T cells also specifically eradicate the primary AML patient-derived blast. In particular, GRP78-CAR T cells spare normal HSCs, highlighting that GRP78-CAR is a promising approach for the therapy of AML.

## Introduction

In recent years, chimeric antigen receptor (CAR) T-cell treatment has achieved great success in clinical trials, especially those in which CAR T cells targeting CD19 have shown excellent response against B-cell lineage hematological malignancies (Scheuermann and Racila, 1995; Porter et al., 2011; Maude et al., 2014; Sommermeyer et al., 2017; Park et al., 2018). However, other subtypes of hematological malignancies, including acute myeloid leukemia (AML) with deficiency of a specific target on the cell surface lack effective therapy.

AML, the most common acute leukemia in adults, is characterized by a clonal expansion of myeloid blasts in the bone marrow, blood, and other tissues. The prognosis of AML patients is still poor, and the 5-year survival rate remains below 50% owing to resistance to the treatments and relapse of the disease. Unlike the B-cell malignancies with antigens that are exclusively expressed in B-cell lineages and B-cell aplasia, which is clinically tolerable, CAR-targeted antigens for myeloid cells are shared with normal hematopoietic stem cells (HSCs); thus, no ideal surface marker can distinguish between normal and tumor cells (Mardiana and Gill, 2020). Several CARs targeting CD33 (Wang et al., 2015) or CD123 (Ruella et al., 2016) show potency in the pre-clinical model; however, these CAR T cells are targeting both normal HSCs and leukemic cells, which lead to myeloablation and bone marrow failure and induce significant cytokine release syndrome (Ehninger et al., 2014; Wang et al., 2015). To prevent the severe side effect induced by the CAR T-cell treatment, several approaches were utilized including limiting the persistence of CAR T cells through engineering a “safety switch” (Di Stasi et al., 2011); novel developed CAR T cells targeting CLL-1 or CD70 are efficient against AML without being toxic to normal HSCs (Tashiro et al., 2017; Wang et al., 2018; Sauer et al., 2021). In the past decades, numerous tumor antigens such as CD33, CD123, CLL1, and CD38 have been explored as target antigens for AML treatment (Jin et al., 2009; Walter et al., 2012; Wang et al., 2018; Cui et al., 2021), and the clinical trials for these CAR T-cell therapies reported improved outcomes. A phase I clinical trial showed that CLL1 CAR T-cell therapy has high efficacy and limited toxicity in relapsed and refractory AML patients (Zhang et al., 2021). Another clinical trial reported that four out of six (66.7%) relapsed patients achieved complete remission (CR) or CR with incomplete count recovery (CRi) after CD38-CAR T-cell infusion, and the clinically adverse effects of patients are manageable (Cui et al., 2021). Since the number of cases in these studies is limited, the safety and efficacy of CAR T-cell therapy for patients with relapsed/refractory AML requires further investigations.

Glucose-regulated protein 78 (GRP78; also known as Bip) as an endoplasmic reticulum (ER) chaperone protein is essential for protein quality control (Quinones et al., 2008). The accumulation of evidence shows that GRP78 contains anti-apoptotic function through activation of UPR and blocking caspase activation to enhance cell survival and contribute to tumor progression (Fu et al., 2007; Casas, 2017). In particular, GRP78 is expressed and located mainly within the ER lumen. However, a small fraction of GRP78 could re-locate to the cell surface on a certain type of cells, particularly tumor cells (Shin et al., 2003; Arap et al., 2004; Gonzalez–Gronow et al., 2009; Zhang et al., 2010). GRP78 on the cell surface of tumor cells promotes cell proliferation, metastasis, and resistance to drug therapy (Lee, 2007; Li and Li, 2012).

A proof-of-concept study reported that the cell surface GRP78 could be specifically targeted by peptidic ligands to induce tumor cell death in prostate and breast cancer models (Arap et al., 2004; Kim et al., 2006). The GRP78 monoclonal antibody MAb159 specifically shows inhibition of tumor cell proliferation and metastasis and leads to tumor regression (Liu et al., 2013). The inhibition of cell surface GRP78 by polyclonal N-20 antibody can suppress glioma cancer cell survival and proliferation (Kang et al., 2016). GRP78 was proven to be highly expressed on the cell surface of AML patients’ peripheral blood cells, and it is also over-expressed in chronic lymphocytic leukemia patients’ cells than in normal B cells, but not in T cells (Huergo-Zapico et al., 2012; Staquicini et al., 2018). Therefore, the overexpression of GRP78 on the plasma membrane of leukemia cells potentially provides a novel target for the therapy of hematological malignancies.

Here, we developed CAR T cells targeting cell surface GRP78 to specifically eradicate AMLs cells *in vitro* and *in vivo*.

## Methods

### Cell Lines and Primary Samples

KG1a, HL-60, and U937 cell lines were from ATCC and were purchased from the China National Collection of Authenticated Cell Cultures and Conservation Genetics CAS Kunming Cell Bank. HL-60 and U937 cells were cultured in RPMI (Gibco) supplemented with 10% FBS, 100 U/ml penicillin, and 100 mg/ml streptomycin sulfate at 37°C with 5% CO_2_. KG1a cells were cultured in RPMI (Gibco) supplemented with 20% FBS, 100 U/ml penicillin, and 100 mg/ml streptomycin sulfate at 37°C with 5% CO_2_. Primary human AML blood samples were obtained from the West China Hospital of Sichuan University with the approval of patients. Blood and bone marrow from healthy donors were provided by the West China Hospital of Sichuan University with the approval of donors (#2022151).

### Chimeric Antigen Receptor T-Cell Production

Primary T cells were isolated from peripheral blood of healthy donors using the RosetteSep Human T Cell Enrichment Cocktail (Stemcell Technologies) according to the manufacturer’s protocol. The isolated T cells were confirmed by flow cytometry using phycoerythrin-conjugated anti-human CD3 (BioLegend, 300408). T cells were cultured in Advanced RPMI1640 (Gibco) supplemented with 10% FBS, 100 U/ml penicillin, 100 mg/ml streptomycin sulfate, and 200 U/ml IL2 (PeproTech). T cells were counted and stimulated by CD3/CD28 beads (Life Technologies) for 72 h and infected with lentiviral particles at an MOI of 100.

### Cytotoxic T Lymphocyte Assay

The specific cytotoxicity of the CAR T cells was measured against the CFSE-labeled target cancer cells at indicated effector/target (E/T) ratios in triplicate wells. After 24 h of culture in Advanced RPMI 1640 (Gibco) supplemented with 10% FBS, 100 U/ml penicillin, and 100 mg/ml streptomycin sulfate, total cells were harvested, and dead cells were labeled with PI following flow cytometry analysis.

### Cytokine Production Assay

Effector cells and target cells were cultured at varied E/T ratios in Advanced RPMI 1640 (Gibco) media for 24 or 48 h. Human interferon gamma (INF-γ; #KHC4021, Invitrogen), tumor necrosis factor (TNF; #550610, BD Biosciences), and granzyme B (#ab235635, Abcam) in the supernatant of culture were analyzed using a commercial assay according to the manufacturer’s instructions.

### Immunofluorescence

A total of 0.5 × 10^6^ cells were collected and washed once with ice-cold PBS (pH 7.4) and incubated with anti-GRP78 (#PA1-014A, Thermo Fisher Scientific; 1:200 in 2% FBS PBS) for 60 min at 4°C. Cells were centrifuged and washed once with PBS followed by fixation in ice-cold methanol (100%) for 10 min at 4°C and washed once again with PBS. Cells were incubated in blocking buffer (2.5% BSA/10% goat serum/0.1% Tween-20) for 30 min at RT. Incubation with goat anti-rabbit IgG (H+L) secondary antibody conjugated with Cyanine3 (#A10520, ThermoFisher Scientific, 1:200) in blocking buffer for 1 h at RT. After washing once with PBS, cell nuclei were stained with DAPI in PBS for 10 min in the dark. The cells were centrifuged and washed once with PBS, resuspended in 30 μl PBS and dropped on the coverslip, mounted with Aqua-Polymount on the slide (#1860620, Polysciences Inc.).

### Xenograft Animal Model

Six-week-old female NOD-Prkdcem26ll2rgem26Nju (NCG) mice were purchased from GemPharmatech Co., Ltd. (Jiangsu, China) and housed in a pathogen-free animal facility. Then, 1.0 × 10^6^ KG1a-Luci or U937-EGFP-Luci cells were intravenously injected into NCG mice *via* the tail vein 1 week after arriving at the local animal facility. Moreover, 5.0 × 10^6^ Non-CAR, MOCK-CAR, and GRP78-CAR T cells were intravenously injected via the tail vein on day 6 for U937-EGFP-Luci–injected mice and day 7 for KG1a-Luci–injected mice after leukemia cells were transplanted, followed by serial bioluminescence imaging to quantify the progression of the tumor. The bioluminescence images were captured using an IVIS imaging system and quantified by Living Image software 4.1 (PerkinElmer). Mice were injected intraperitoneally with Avertin (375 mg/kg) and D-luciferin (150 mg/kg) and imaged under anesthesia. Mice were sacrificed when they were in the moribund state or when they showed signs of hind limb paralysis or at end of the experiment time point (50 days post leukemia cell injection for U937-EGFP-Luci or 100 days for KG1a-Luci).

### Flow Cytometry

Primary AML cells were isolated through density gradient centrifuge, and then the remaining red blood cells were lysed. For the cell lysis analysis of CD34^+^ progenitor/stem cell subsets, CD34-FITC mAbs (Clone 4H11, Invitrogen) were stained, and the positive subset was analyzed using the percentage of lysis after staining with PI. The transduction rate of CAR into T cells was detected using mKate signal. Flow cytometry was performed on a BD Fortesa flow cytometer, and results were analyzed using the software FlowJo v10.

## Results

### Glucose-Regulated Protein 78–Chimeric Antigen Receptor T Cells Demonstrated Robust Cytotoxicity Against Leukemia Cells *In Vitro*

**FIGURE 1:**
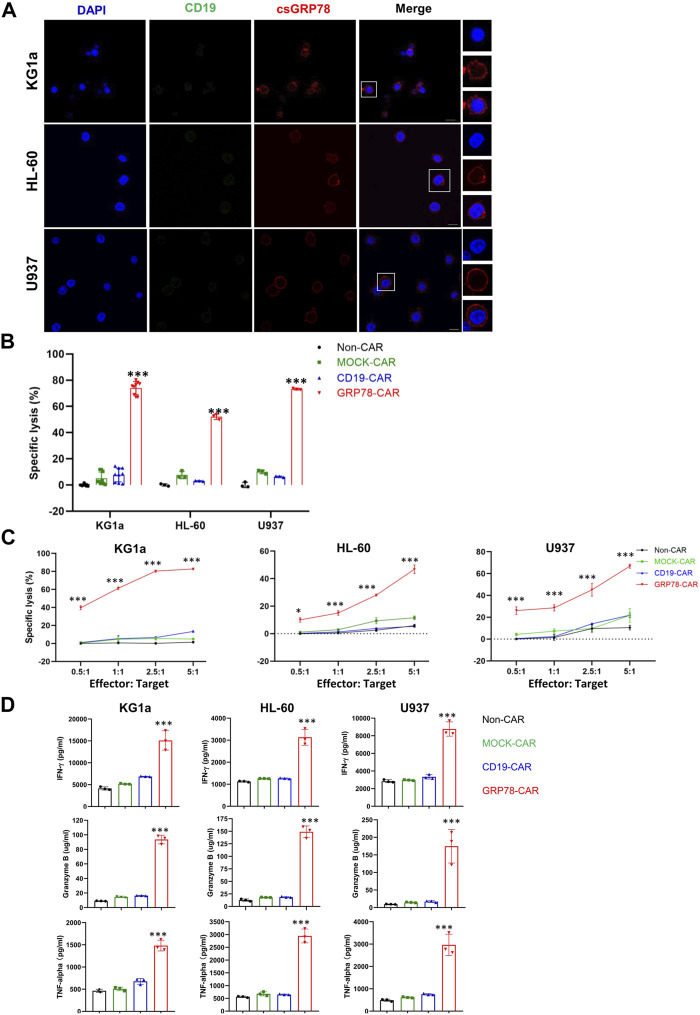
Glucose-regulated protein 78 (GRP78)–chimeric antigen receptor (CAR) T cells demonstrate anti-leukemic effect against acute myeloid leukemia cells. **(A)** Confocal image of representative immunofluorescence staining of cell surface GRP78 (red) and CD19 (green) on indicated leukemia cell lines. The denoted cells are zoomed on the right. The bars indicated 10 μm length. **(B)** cytotoxic assay measuring the specific lysis of target cells. CFSE-labeled AML cell lines KG1a, HL-60, and U937 cells were co-incubated with Non-CAR T, MOCK-CAR T, CD19-CAR T, and GRP78-CAR T cells at an effector/target (E/T) ratio of 5:1 for 24 h. Experiments were repeated with at least triplicate samples. Data represent means ± SDs. Two-sided Student’s t-test was performed between GRP78-CAR and Non-CAR, MOCK-CAR, and CD19-CAR, ***p < 0.01. **(C)** leukemia cells were incubated with Non-CAR T, MOCK-CAR T, CD19-CAR T, and GRP78-CAR T cells at various (E/T) ratios as indicated. Percentages of lysis cells are shown as means ± SDs. All experiments were repeated with at least triplicate samples. GRP78-CAR vs*.* Non-CAR, MOCK-CAR, and CD19-CAR T cells with the two-sided Student’s t-test, ***p < 0.01. **(D)** ELISA measurements of cytokines interferon gamma (IFN-γ), tumor necrosis factor–α (TNF-α), and granzyme B in the cell supernatant were determined following 24-h incubation of target cells with specified CAR-T cells. Two-side Student’s t-test was performed between GRP78-CAR T cell treatment and Non-CAR, MOCK-CAR, and CD19-CAR T cells. All experiments were repeated with at least triplicate samples. Data represent means ± SDs. ***p < 0.01.

To access the expression of GRP78 on the cell surface, we performed live-cell staining with GRP78, observing enhanced GRP78 expression on the plasma membrane of multiple AML cell lines (Figure 1A; Supplementary Figure S1). Thus, the enriched GRP78 on the cell surface provides the opportunity to be targeted.

We constructed a Pep42 peptide into a second-generation CAR construct to generate GRP78-CAR that contains 4-1BB and CD3ζ costimulatory signaling domains (Supplementary Figure S2A). Healthy T cells were expanded and transduced by a GRP78-CAR lentivirus, and Non-CAR, MOCK-CAR, and CD19-CAR were used as controls. The CAR lentivirus transduction is efficient in that more than 50% of T cells expressed CAR (Supplementary Figure S2B). The cytotoxicity of CAR T cells was assessed by co-culturing with leukemia cells *in vitro* for 24 h. It was observed that GRP78-CAR T cells robustly elicited specific cytotoxicity against all three AML cell lines (Figure 1B; GRP78-CAR vs*.* Non-CAR, MOCK-CAR, and CD19-CAR). Next, we evaluated the dose-dependent cytotoxicity of the CAR T cells by progressively increasing the E/T ratio from 0.5:1 to 5:1. GRP78-CAR T cells exhibited robust lysis against AML cells, even at the 0.5:1 ratio for KG1a and U937 cells (Figure 1C). Considerable amounts of released cytokines including IFN-γ, TNF-α, and granzyme B were detected in the supernatant of the medium after GRP78-CAR T cells were co-cultured with AML cells (Figure 1D). Together, the result of the *in vitro* cytotoxicity assay provides compelling evidence that GRP78-CAR T cells can effectively kill AML cells *in vitro*.

### Glucose-Regulated Protein 78–Chimeric Antigen Receptor T Cells Eliminate Human Acute Myeloid Leukemia *In Vivo* in Xenograft Mouse Models

**FIGURE 2:**
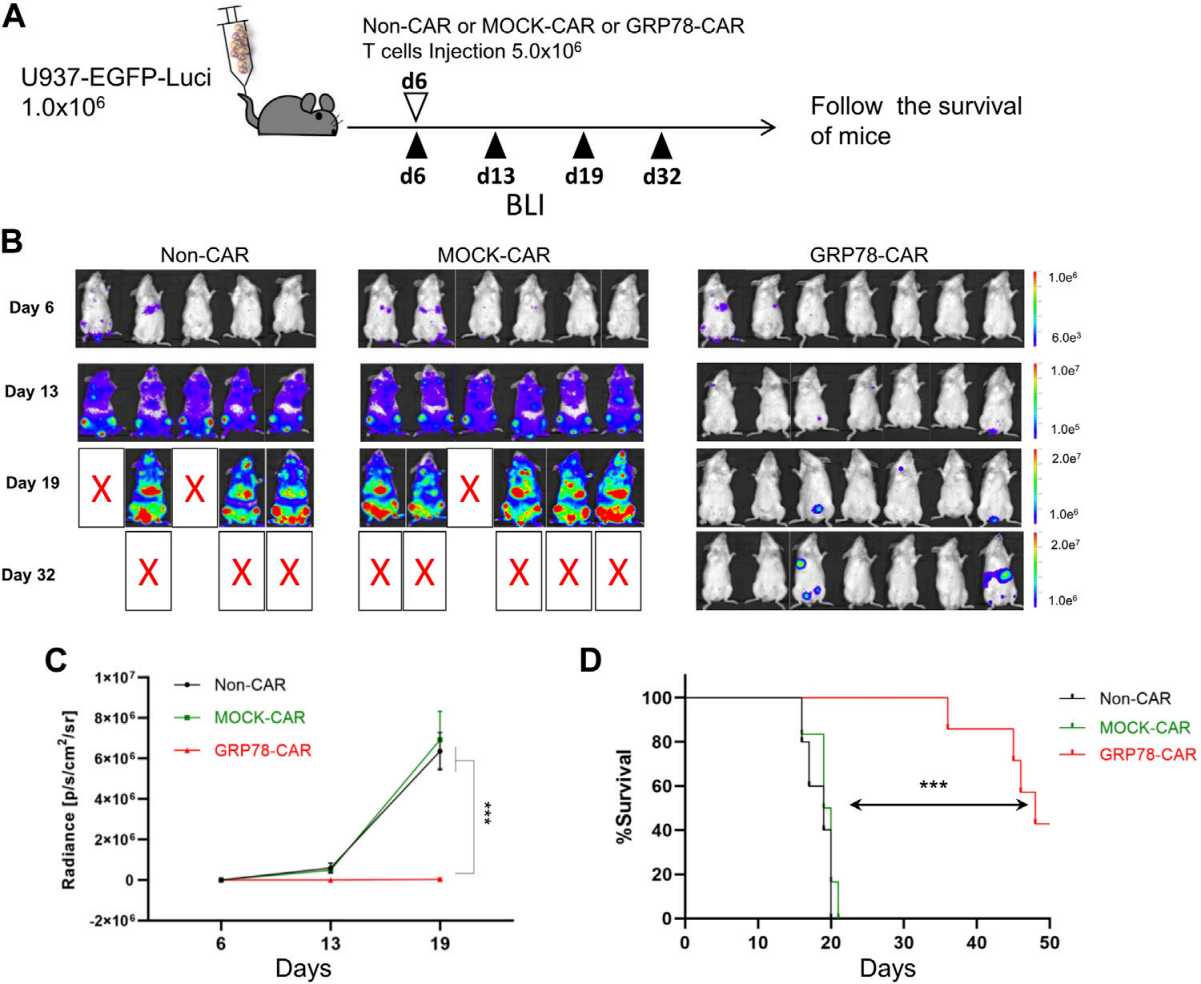
Glucose-regulated protein 78 (GRP78)–chimeric antigen receptor (CAR) T cells eliminate human acute myeloid leukemia (AML) *in vivo* in xenograft mouse models. **(A)** Schematic of the U937 leukemia cell xenograft model. NSG mice were injected *via* the tail vein with 1 × 10^6^ U937-EGFP luciferase (U937-EGFP-Luc) on day 0. Bioluminescent imaging (BLI) was performed on day 6 to quantify engraftment and for randomization of treatment groups. GRP78-CAR T cells (5  ×  10^6^), MOCK-CAR T cells (5  ×  10^6^), or Non-CAR T cells (5  ×  10^6^) were injected via the tail vein on day 6, followed with serial BLI. BLI radiance was measured as a surrogate quantification of tumor burden. **(B)** tumor burden was visualized by BLI on days 6, 13, and 19 following U937-EGFP-Luc cell transplantation. **(C)** bioluminescent signal for each treatment group over time. Data represent mean values of each group ± SEMs. The number of mice in each group is listed in **(B)**. Two-way ANOVA test was performed between GRP78-CAR T-cell treatment and Non-CAR and MOCK-CAR T cells. ***p < 0.01. **(D)** Kaplan–Meier analysis of survival. Log-rank (Mantel–Cox) tests were used to perform statistical analyses of survival between groups.

The anti-leukemia cytotoxicity *in vivo* was validated through the human AML xenograft mouse model, in which 1 × 10^6^ U937-EGFP-Luci leukemia cells were intravenously injected into NSG mice, followed by 5.0 × 10^6^ Non-CAR T cells, MOCK-CAR T cells, and GRP78-CAR T cells injection at day 6 (Figure 2A). The burden of tumor cells was monitored over a series of days by luminescence imaging.

The mice injected with Non-CAR T cells and MOCK-CAR T cells showed first signs of disease, such as reduced mobility, hypothermia, and scrubby fur, within 17 days post injection, strikingly compared to 34 days for the mice injected with GRP78-CAR T cells (Figure 2B). Control mice that received Non-CAR T or MOCK-CAR T-cell injection displayed rapid progression of leukemia that 50% of animals succumbed 19 days post leukemia cell injection. In sharp contrast, among GRP78-CAR T-cell–treated mice, 50% survived more than 45 days after leukemia cell injection (Figure 2D). Moreover, three out of seven mice survived healthy for 50 days after tumor cell challenges, and tumor burden imaging confirmed that the vast majority of the leukemia cells were eliminated *in vivo* (Figures 2C,D).

We also tested the therapeutic efficacy of GRP78-CAR T cells by intravenous injection of AML KG1a-Luci cells into NSG mice, and the groups of mice treated with Non-CAR T cells, MOCK-CAR T cells, or GRP78-CAR T cells were monitored by bioluminescence at indicated days post injection (Supplementary Figure S3A). The AML burden in mice was quantified and surrogated as bioluminescence, and the group of mice with GRP78-CAR T-cell injection exhibited strikingly lower bioluminescence signal than mice treated with Non-CAR T cells and MOCK-CAR T cells, indicating that the vast majority of KG1a leukemia cells were eradicated by GRP78-CAR T cells *in vivo* (Supplementary Figures S3B,C). After analysis of lymphoid organs of sacrificed mice, the residual leukemia cells in lymphoid organs are rarely detectable in mice injected with GRP78-CAR T cells (Supplementary Figures S3D,E).

Altogether, the *in vivo* AML xenograft mouse experiment is consistent with the *in vitro* cytotoxicity assay that GRP78-CAR T cells can eliminate leukemia cells both *in vitro* and *in vivo*.

### Glucose-Regulated Protein 78–Chimeric Antigen Receptor T Cells Eradicated Primary Acute Myeloid Leukemia Blast

**FIGURE 3:**
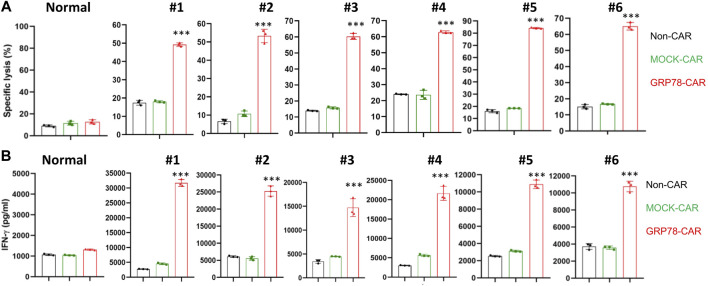
Glucose-regulated protein 78 (GRP78)–chimeric antigen receptor (CAR) T cells eradicate primary acute myeloid leukemia (AML) blasts. **(A)** cytotoxic assay measuring the specific lysis of target cells. CFSE-labeled primary AML patient PBMC were co-incubated with Non-CAR T, MOCK-CAR T, and GRP78-CAR T cells at an effector/target ratio of 5:1 for 48 h. The normal PBMC sample was collected from the blood of the healthy donors. Experiments were repeated with at least triplicate samples. Data represent means ± SDs. GRP78-CAR vs*.* Non-CAR, MOCK-CAR, and CD19-CAR. ***p < 0.01 (two-sided Student’s t-test). **(B)** ELISA measurement of cytokine interferon (IFN) gamma in the cell supernatant was determined following 48-h incubation of primary PBMC cells with specified CAR T cells. Two side Student’s t-test was performed between GRP78-CAR T cell treatments with Non-CAR and MOCK-CAR. Data represent means ± SDs. GRP78-CAR vs*.* Non-CAR, MOCK-CAR, CD19-CAR. ***p < 0.01 (two-sided Student’s t-test).

Next, we determined whether GRP78-CAR T cells could elicit cytotoxicity against primary AML blasts. A panel of normal and primary AML blood samples obtained from healthy donors and AML patients were co-cultured with CAR T cells at an E/T ratio of 10:1 for 48 h. Cytotoxicity induced by CAR T cells against normal or patients’ samples *in vitro* was determined by cytotoxic T lymphocyte assay. In total, 24 patients’ blood samples were collected, and the fraction of blasts in peripheral blood cells varied from 18% to 99% including variable FAB subtypes (Supplementary Table S1). GRP78-CAR T cells induced significantly higher cytotoxicity against AML blast samples (6 out of 24, 25%), but no cytotoxicity to normal samples (Figure 3A). In contrast to control CAR T cells, GRP78-CAR T-cell treatments released significantly higher levels of cytokine IFN-γ to the supernatant, which indicates that GRP78-CAR T cells specifically elicit immune cytotoxicity to patients’ AML blasts (Figure 3B). Furthermore, immunofluorescence staining was performed on GRP78-CAR T-cell response samples and confirmed that GRP78 is enriched on the plasma membrane of patients’ tumor samples (Supplementary Figure S4).

### Hematopoietic Stem Cells Are Spared by Glucose-Regulated Protein 78–Chimeric Antigen Receptor T Cells

**FIGURE 4:**
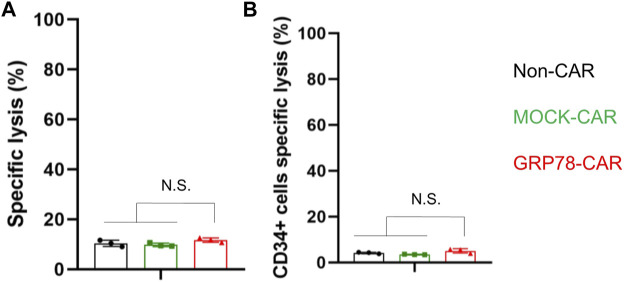
Hematopoietic stem cells are spared by glucose-regulated protein 78 (GRP78)–chimeric antigen receptor (CAR) T cells. **(A)** cytotoxic assay measuring the specific lysis of bone marrow cells from healthy donors. CFSE-labeled primary normal bone marrow cells were co-incubated with Non-CAR T, MOCK-CAR T, and GRP78-CAR T cells at an effector/target ratio of 5:1 for 24 h. Experiments were repeated with at least triplicate wells. Data represent means ± SDs. ***p < 0.01 (two-sided Student’s t-test). **(B)** cytotoxicity of Non-CAR, MOCK-CAR, and GRP78-CAR T cells against hematopoietic stem cells (CD34^+^) derived from the bone marrow of healthy donors. The cells were co-cultured at an effector/target ratio of 5:1 for 24 h. Data represent mean values of triplicate wells ± SDs. ***p < 0.01 (two-sided Student’s t-test).

The limitation of CAR T-cell treatment for AML is because of the lack of a tumor-specific antigen enriched in leukemia cells but not in normal cells and HSCs. Therefore, we isolated cells from the bone marrow of healthy donors after lysis of red blood cells to co-incubate with CAR T cells. GRP78-CAR T cells exhibit a comparable level of cytotoxicity with Non-CAR and MOCK-CAR T cells (Figure 4A). Next, the cytotoxicity of engineered CAR T was tested on the HSCs (CD34^+^) derived from the bone marrow of healthy donors. After 24 h of co-culture of CAR-T cells with the cell mixture, no increment of cell lysis was observed on the CD34^+^ cell subpopulation (Figure 4B). Another sample from healthy donors showed a consistent result (Supplementary Figure S5). Overall, stem cells derived from the hematopoietic system are not targeted by GRP78-CAR T cells, suggesting that the engineered GRP78-CAR T cell is a safe therapy for AML without the potential toxicity.

Regarding CAR lentivirus transfection that may lead to CAR T cells fratricide via self-targeting, we tested the GRP78-CAR T cells’ induced cytotoxicity to primary T cells. It was confirmed that no significant cytotoxicity was induced by GRP78-CAR T cells in comparison to control CAR T cells and accompanied by a similar level of cytokine production (Supplementary Figures S6A,B). GRP78-CAR lentivirus transfection also exhibits no impairment to the T-cell proliferation (Supplementary Figure S6C), which facilitates the expansion and preparation of GRP78-CAR T cells *in vitro*. Furthermore, GRP78 was barely detected on the cell surface of normal T cells by immunostaining, which is consistent with the above observation (Supplementary Figure S6D).

## Discussion

AML is the most common acute leukemia in adults. Although for AML patients, CR is achieved in 60–80% of younger adults and 40–60% of older adults after standard chemotherapy, in the majority of cases, AML will relapse within 3 years (Döhner et al., 2010; Döhner et al., 2015; Döhner et al., 2017). CAR T-cell therapy is an emerged technology that is developed and proven to be an effective treatment for hematological malignancies, such as CAR T cells targeting CD19 for B-cell malignancies. However, the lack of effective CAR-T for the therapy of AML is because of the short of specificity. Previous studies reported that CAR-T cells targeted CD33/CD123, which antigens targeted both leukemia cells and hematological stem cells, indicating CD33/CD123 CAR-T treatment might result in the bone marrow failure (Pizzitola et al., 2014).

Current CAR T cells targeting CLL-1 and CD70 demonstrated cytotoxicity to tumor cells and spared CD34^+^ stem cells, indicating the potency of clinical implications for these two CAR T-cell treatments. However, it is well known that AML is featured with genetic heterogeneity and variable fusion genes as tumorigenesis drivers. Only a fraction of AML cells expresses CLL-1 or CD70 on the plasma membrane as the target for CAR T cells. Furthermore, relapse after CAR T-cell therapy remains a challenge owing to the mutation or down-regulation of the antigen on the tumor cell surface, which leads to the tumor escaping from CAR targeting and consequently results in resistance to therapy (Ruella and Maus, 2016; Shah and Fry, 2019). To overcome tumor antigen escape, the CAR is designed to simultaneously target multiple cell surface antigens, such as CD22 and CD20 (Zah et al., 2016; Fry et al., 2018). Therefore, it underscores the necessity of developing novel CAR T cells that target diverse antigens to combine or complement the current CAR T-cell therapy for AML and provide more options for patients.

GRP78, a chaperone protein that belongs to the heat-shock protein family, plays a crucial role in maintaining cellular homeostasis. It is an evolutionarily conserved protein that presents in multiple subcellular positions and exerts distinct functions: retains in the ER to produce unfolded protein response, binds to mitochondria to interact with apoptotic executors, and resides in the plasma membrane to transduce proliferative signal as a receptor (Zhang and Zhang, 2010). The plasma membrane GRP78 has been uncovered in various human cancers, including leukemia (Staquicini et al., 2018), melanoma (de Ridder et al., 2011), prostate cancer (Arap et al., 2004), and colorectal cancer (Shin et al., 2003; Li et al., 2013). In the tumor cells, the cell surface GRP78 has a crucial role in protection from apoptosis, promotion of proliferation, evasion from immune surveillance, and resistance to various therapies (Gonzalez–Gronow et al., 2009; Zhang and Zhang, 2010). However, the mechanisms by which GRP78 is translocated to the cell surface remain elusive. Noting that the tumor cells exhibit a high level of GRP78 on the plasma membrane, targeting GRP78 to induce cytotoxicity of tumor cells becomes an attractive method. Several studies designed GRP78-binding peptides conjugated with cytotoxic drugs to precisely target cancer cells, which demonstrated promising results *in vitro* and *in vivo* (Arap et al., 2004; Kim et al., 2006; Yoneda et al., 2008). Jiang et al. (2019) constructed GRP78-targeted nanocage to specifically target and kill hepatocellular carcinoma and suppress lung metastasis. Here, we cloned Pep42, a cyclic oligopeptide that specifically targets cell surface GRP78, into a second-generation CAR construct to generate GRP78-CAR and demonstrated that GRP78-CAR T cells can effectively induce cytotoxicity of AML cells *in vitro* and eradicate explanted leukemia cells *in vivo*. Furthermore, in a cohort of 24 AML patients, we found that GRP78-CAR T cells effectively eradicated AML blasts in a fraction of AML specimens (25%) with different FAB subtypes, supporting its promising application for AML treatment.

In normal cells, previous studies reported that the functions of GRP78 are binding to polypeptides in the ER and activating unfolded protein response when polypeptides are overproduced. Thus, GRP78 resides in the lumen of the ER to maintain intracellular homeostasis. Besides, several studies have uncovered that GRP78 can also express on the plasma membrane of various cancer cells and endothelial cells (Davidson et al., 2005; Lee, 2007; Gonzalez–Gronow et al., 2009). Therefore, GRP78 serves as a suitable target for CAR T-cell therapy. In principle, CAR T cells targeting the cell surface of GRP78 have the advantage of sparing normal cells and specifically inducing cytotoxicity of tumor cells. In particular, for the therapy of AML, which requires greater cancer cell specificity, in pioneer studies, CAR T cells targeting antigens CD33, CD44, and CD123 have been developed (Jin et al., 2006; Jin et al., 2009; Walter et al., 2012). However, these treatments exhibit side effects when normal HSCs are targeted accompanied by bone marrow toxicity, which leads to myeloablation. Our result supports that GRP78-CAR T cells are not targeting the normal cells and avoid cytotoxicity to HSCs, which ensures the safety of GRP78-CAR T-cell therapy.

Aligned with our study, a recent study by Hebbar et al. (2022) also reported that GRP78 is a promising target for CAR T cells in the therapy of AML. Both studies highlighted that GRP78-CAR T cells are able to eradicate the malignant cells *in vitro* and *in vivo* and the same peptide (Pep42) was chosen to construct CAR. Both studies confirmed that GRP78-CAR T cells are safe and would not impair the HSCs. In our study, we confirmed that 6 out of 24 fresh AML primary blasts can be eradicated by GRP78-CAR T cells, while they measured the expression of GRP78 in 14 primary AML samples without testing the cytotoxicity of GRP78-CAR T cells. Thus, we might provide more direct evidence of GRP78-CAR T cells’ induced cytotoxicity to primary AML samples. In their study, flow cytometry was performed and demonstrated that GRP78 is expressed on the cell surface of more than half of primary AML samples using an antibody for ER retention sequence (KDEL) and a biotin-conjugated peptide, but the antibody used in the study is no longer supplied by the company. In our study, we performed immunostaining to assess the expression and visualize the localization of GRP78 for living AML cells and primary samples. We observed that GRP78 is expressed on the plasma membrane of three AML cell lines and 25% of primary AML samples, which is consistent with the cytotoxicity induced by GRP78-CAR T cells. The lower percentage of GRP78 we detected might be because of the difference in antibodies, more stringent criteria used in immunofluorescence staining, and the sizes of samples. Furthermore, the GRP78-CAR T cells generated in our study might be more effective than those generated by Hebbar et al. (2022). Their study proved the anti-AML activity of GRP78-CAR T cells *in vivo* by injection of 5 × 10^3^ MOLM13 AML cells following 3 × 10^6^ CAR T cells on day 7, whereas our study demonstrated that 5 × 10^6^ GRP78-CAR T cells can eradicate explanted 1 × 10^6^ of U937 or KG1a AML cells in the xenograft mouse models. Together, both studies uncover the anti-AML activity of GRP78-CAR, thus highlighting GRP78-CAR T-cell therapy as a promising approach in the clinic.

In this study, we constructed the GRP78-CAR and demonstrated that GRP78-CAR T cells manifested high cytolytic effects against AML cells *in vitro* and significantly eradicated the tumor in xenograft mouse models. Moreover, GRP78-CAR T cells could notably eliminate primary AML blasts, which indicates a substantial fraction of AML patients could benefit from GRP78-CAR T-cell therapy in the future. GRP78-CAR T cells showed no cytotoxicity on normal bone marrow cells and HSCs. Together, GRP78-CAR T cells can effectively eradicate AML and have the potency to be applied in clinical therapy.

## Data Availability

The original contributions presented in the study are included in the article/Supplementary Material, and further inquiries can be directed to the corresponding authors.
