# Supplementary material for: Chimeric Antigen Receptor T Cells Targeting Cell Surface GRP78 to Eradicate Acute Myeloid Leukemia
Source: Front Cell Dev Biol. 2022 Aug 4;10:928140. doi: 10.3389/fcell.2022.928140 (PMC9387679; doi:10.3389/fcell.2022.928140)
Supplement: Supplementary file 1 [file DataSheet1.PDF]

Supplementary Figure S1

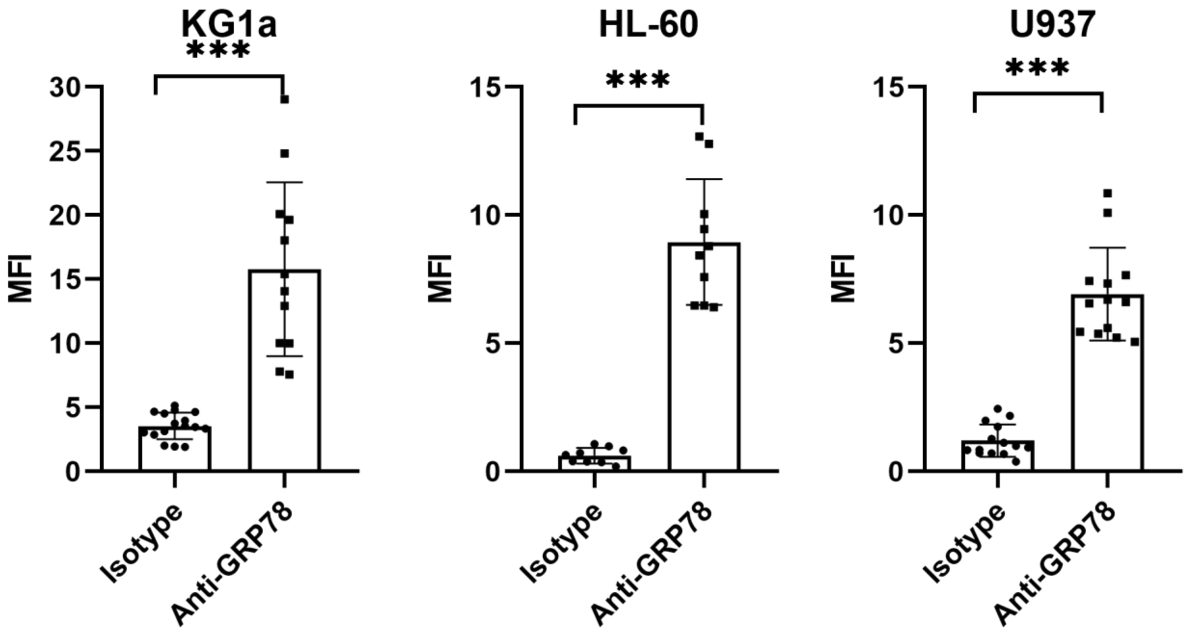

Figure S1.

The mean fluorescence intensity of cell surface GRP78 was quantified for acute myeloid leukemia cells. Each dot represented a single cell. Data represent means ±SD (Two-sided Student’s t-test, ***p<0.01).

Supplementary Figure S2

A

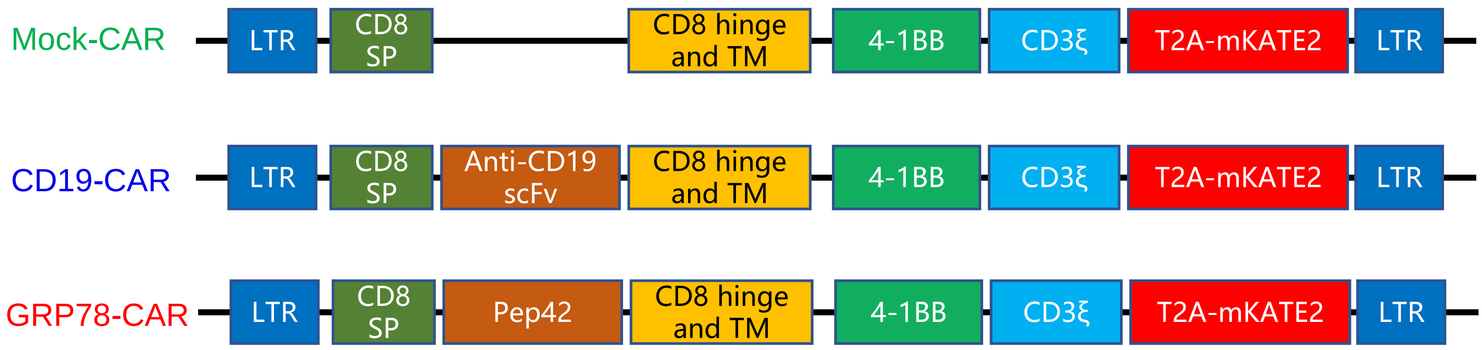

B

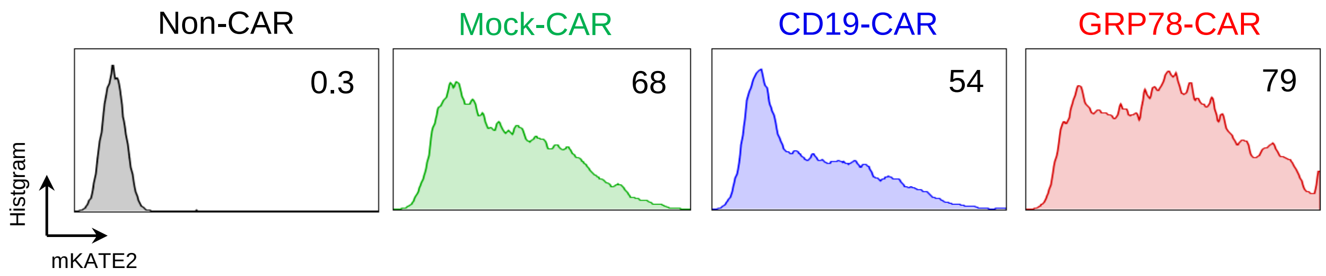

Figure S2.

A Schematic of CAR constructions.

B The representative of flow cytometer measurement of CAR lentivirus transduction efficacy. The efficacy were represented by mKATE2 intensity and indicated on top right.

Supplementary Figure S3

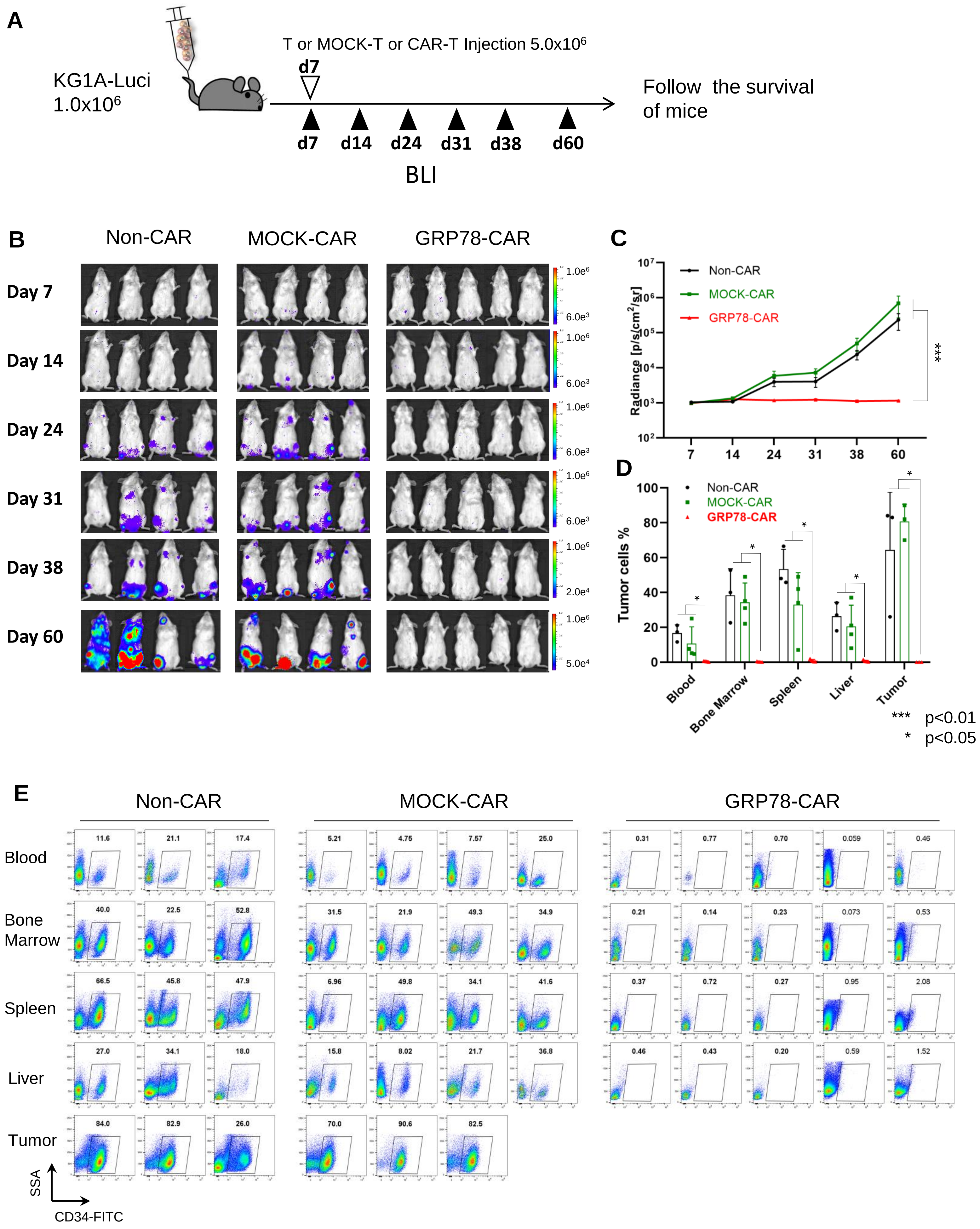

Figure S3.

A. Schematic of the KG1a leukemia cells xenograft model. NSG mice were injected via tail vein with 1×10^6 KG1a luciferase cells (KG1a-Luc) on day 0. Bioluminescent imaging (BLI) was performed on day 7 to quantify engraftment and for randomization of treatment groups. GRP78-CAR T cells (5×10^6), Mock-CAR T cells (5×10^6), or Non-CAR T cells (5×10^6) were injected via tail vein on day 7, and following with serial BLI. BLI radiance was measured as a surrogate quantification of AML burden.

B. Tumor burden were visualized by bioluminescent imaging on day 7 ,14, 24, 31,38,60 following KG1a-Luc transplantation.

C. Bioluminescent signal for each treatment group over time. Data represent mean values of each group ± SD. The number of mice in each group is listed in B. Data represent means ±SD (Two-sided Student's t-test). ***P < 0.01 (Two-way ANOVA test).

D. Quantification of leukemia cells in lymphoid organs. Data represent means ±SD (Two-sided Student's t-test). *P < 0.05 (Two-sided Student's t-test).

E. Representative plots of lymphoid organs obtained from sick mice injected with KG1a-Luc cells. Numbers in the top of square indicate the percentage of KG1a leukemic cells among live single cells. Injected KG1a cells were stained with CD34-FITC antibody.

Supplementary Figure S4

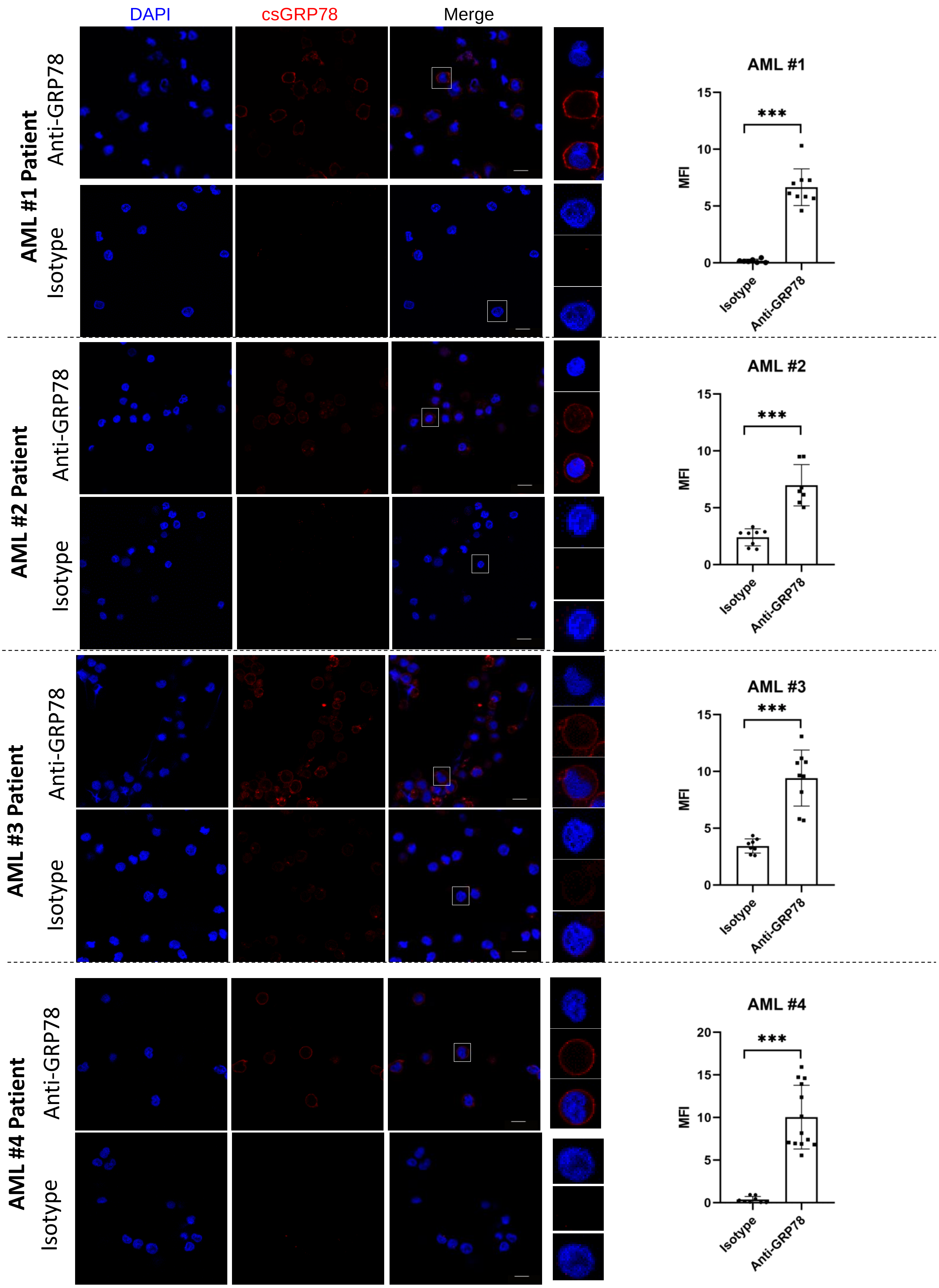

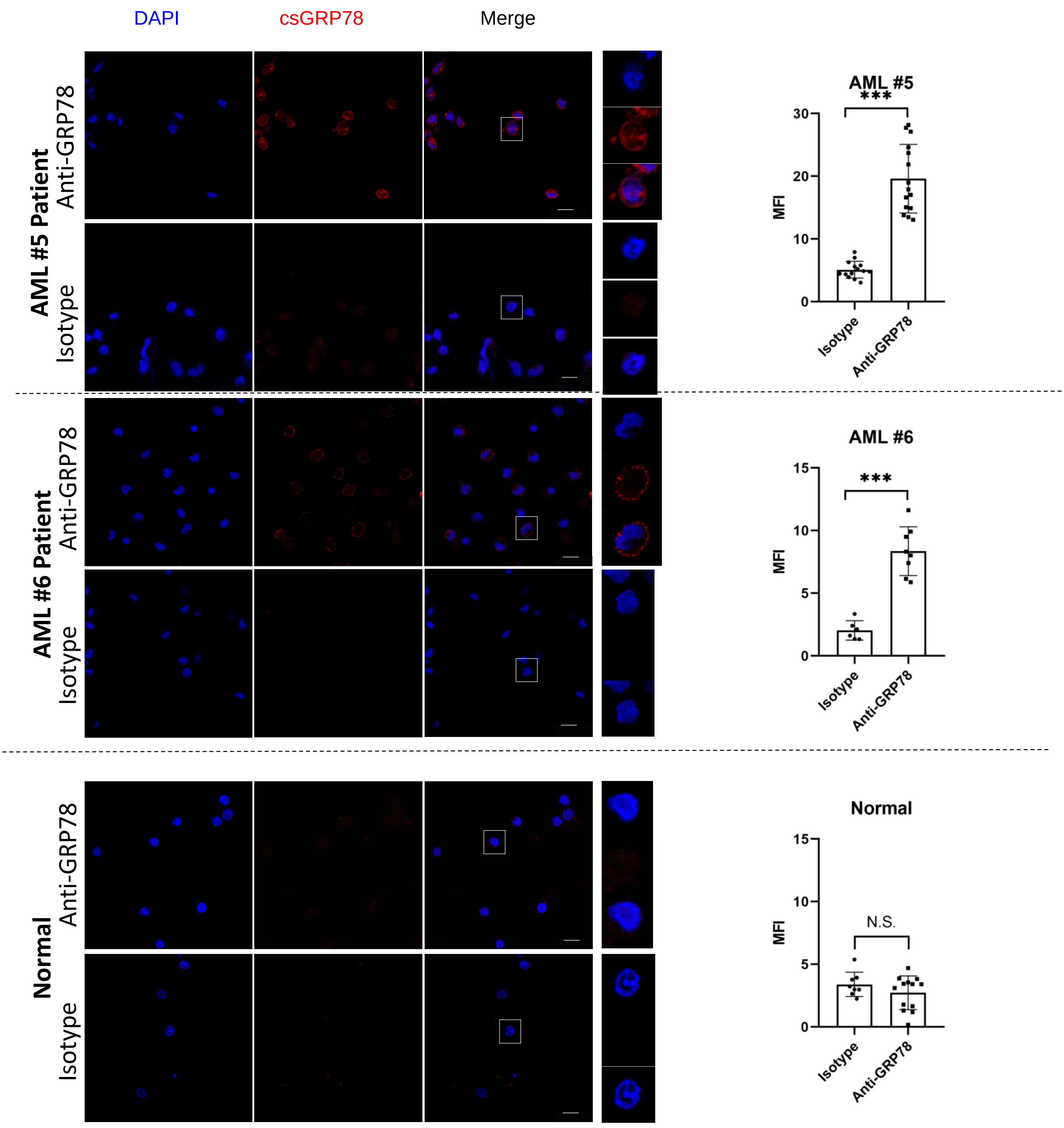

Figure S4.

Confocal image of representative immunofluorence staining of cell surface GRP78 (red) and DAPI (blue) on indicated primary AML patients' PBMC. The denoted cells are zoomed in the right. The bars indicated 10μm length. The mean fluorescence intensity of cell surface GRP78 was quantified by Image J and presented on right panel. Each dot represented a single cell. Data represent means ±SD (Two-sided Student's t-test, ***p<0.01).

Supplementary Figure S5

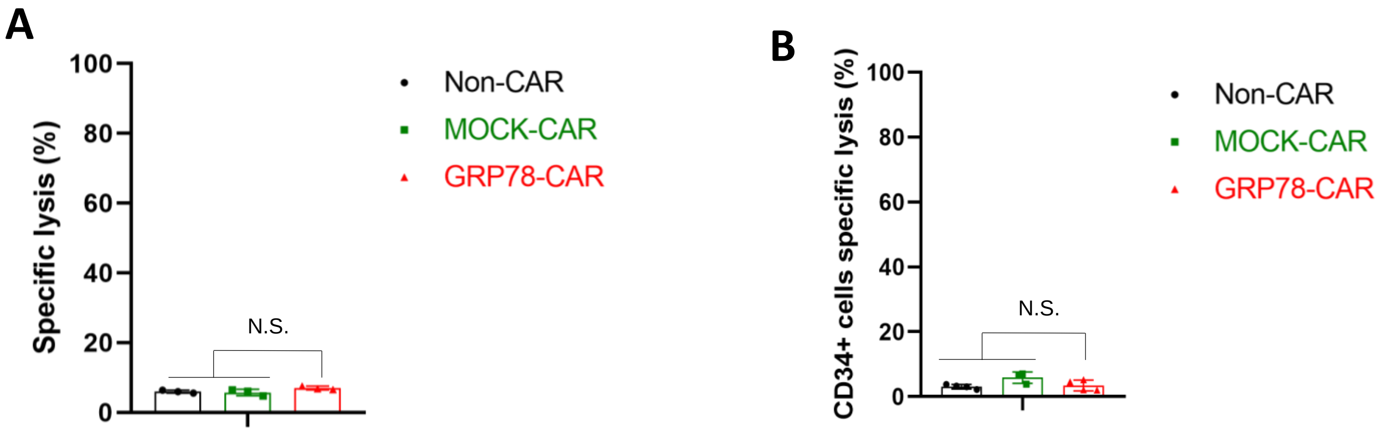

Figure S5.

Cytotoxicity of CAR-T cells to bone marrow cells (A) and hematopoietic stem cells (B) from healthy donors. CFSE-labeled bone marrow cells were co-incubated with Non-CAR T, MOCK-CAR T, and GRP78-CAR T cells at effector:target ratio 5:1 for 24 hours. Hematopoietic stem cells were labeled with CD34-FITC antibody and lysis cells were quantified by PI staining and effector:target ratio is 5:1 and co-incubated for 24 hours . Experiments were repeated with at least triplicate wells. Data represent means ±SD (Two-sided Student's t-test).

Supplementary Figure S6

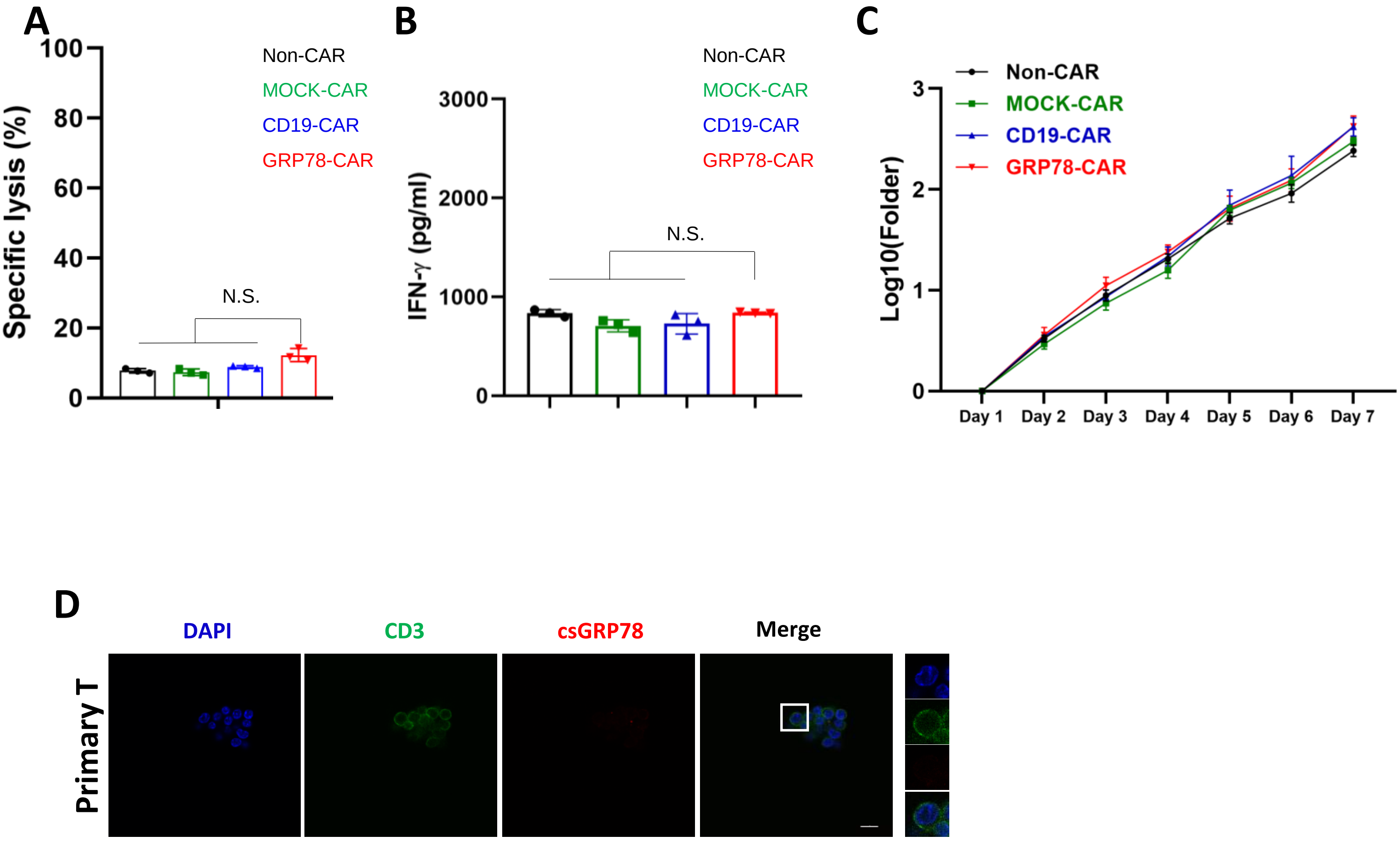

Figure S6.

A. Cytotoxicity of CAR-T cells to primary T cells. CFSE-labeled primary T cells were co-incubated with Non-CAR T, MOCK-CAR T, CD19-CAR T and GRP78-CAR T cells at effector:target ratio 5:1 for 24 hours. Experiments were repeated with at least triplicate samples. Data represent means ±SD. ***$P < 0.01$ (Two-sided Student's t-test).

B. The quantification of IFN-gamma production by ELISA.

C. Measurement of cell growth in indicated days after CAR lentivirus infection.

D. Confocal image of representative immunofluorence staining of cell surface GRP78 (red) and DAPI (blue) on normal primary T cells.

Supplementary Table S1. AML patients' information

No.	Sample source	Sex	Age	Status	Subtype	WBC($x10^9$)	Blast %	Flow result list
1	PB	Female	79	New diagnosed	M4	30.54	35	HLA-DR+,CD123+,CD117(Dim)+,CD11b(Dim)+,CD13+,CD15(Dim)+,CD33+,CD36(Dim)+,CD64(Dim)+,cMPO(Dim)+,CD7+,CD14(Dim)
2	PB	Female	53	New diagnosed	M5	153.74	68	CD117(Dim)+,HLA-DR(Dim)+,CD123+,CD13(Dim)+,CD33(Dim)+,CD64(Dim)+,CD7(Dim)+,MPO(Dim)
3	PB	Male	41	New diagnosed	M3	9.68	49	CD34(Dim)+,HLA-DR(Dim)+,CD117+,CD123+,CD13+,CD15(Dim)+,CD33+,CD64(Dim)+,cMPO+,CD56+
4	PB	Female	44	New diagnosed	M5	12.5	56	HLA-DR+,CD123+,CD117+,CD11b(Dim)+,CD13(Dim)+,CD15+,CD33+,CD36(Dim)+,CD56+,CD64(Dim)+,CD14(Dim)
5	PB	Female	37	No remission	M3	10.91	69	Unknown
6	PB	Female	24	New diagnosed	M3	94.58	99	Unknown
7	PB	Male	23	New diagnosed	M5	2.48	75	CD34(Dim)+, HLA-DR+, CD117+, CD123+, CD13+, CD15+, CD33+, CD38+, CD64+
8	PB	Male	67	New diagnosed	M4	9.78	33	CD38+, CD34+, HLA-DR+, CD123+, CD117+, CD13+,CD15+,CD33+,CD36+,CD64+,cMPO+
9	PB	Female	73	New diagnosed	M1	15.35	92	CD38+,CD117+,CD123+,CD13+,CD15(Dim)+,CD33+,CD64(Dim)+,cMPO+
10	PB	Male	54	No remission	M1	1.53	66	CD34+,HLA-DR+,CD117+,CD38+,CD56+,CD33(Dim)
11	PB	Female	23	New diagnosed		1.8	42	CD34+,HLA-DR+,CD117+,CD123+,CD33+,CD11b(Dim)+,CD13(Dim)+,CD19(Dim)+,CD64(Dim)+,CD56(Dim)
12	PB	Male	20	New diagnosed	M3	7.09	47	CD117+,CD123+,CD13+,CD33+,CD56(Dim)+,CD64+
13	PB	Male	34	New diagnosed		7.13	60	CD34+,HLA-DR+,CD117+,CD123(Dim)+,CD13(Dim)+,CD15(Dim)+,CD33(Dim)+,CD38+,CD56+,CD64(Dim)+,cMPO(Dim)+,CD19(Dim)
14	PB	Male	24	New diagnosed	M3	3.56	18	CD117+,CD123+,CD13+,CD33+,CD64+
15	PB	Female	33	New diagnosed	M2	44.73	81	CD34(Dim)+,CD117+,CD123+,CD13(Dim)+,CD33+,CD38+,cMPO(Dim)
16	PB	Female	49	New diagnosed	M2	34.35	89	CD34+,HLA-DR+,CD117+,CD123+,CD13+,CD33(Dim)+,CD38+,CD19(Dim)+,CD56+,CD64(Dim)+,cMPO(Dim)
17	PB	Female	33	New diagnosed	M2	29.97	66	HLA-DR+,CDCD7+,CDCD13+,CDCD15+,CDCD33+,CDCD34+,CDCD38+,CDCD58+,CDCD117+,MPO+
18	PB	Male	50	New diagnosed		3.14	51	CD34+,HLA-DR+,CD117+,CD123(Dim)+,CD13+,CD33(Dim)+,CD38+,cMPO(Dim)
19	PB	Female	42	New diagnosed	M1	6.68	76	HLA-DR+,CD123+,CD117+,CD13+,CD15(Dim)+,CD33+,CD38+,CD36+,cMPO(Dim)+,CD7+
20	PB	Male	62	New diagnosed		80.54	71	CD38+,CD34+,HLA-DR+,CD117(Dim)+,CD13+,CD33(Dim)+,CD64(Dim)+,cMPO(Dim)+,CD19+,CD10(Dim)+,CD20(Dim)+,CD22+,CD5(Dim)
21	PB	Male	18	New diagnosed	M2	8.04	45	CD38(Dim)+,CD34+,HLA-DR+,CD117+,CD123+,CD13(Dim)+,CD33+,cMPO(Dim)+,CD19(Dim)+,CD56(Dim)+,CD64(Dim)
22	PB	Male	25	New diagnosed	M2	3.33	69	CD34+,HLA-DR+,CD117+,CD123(Dim)+,CD13+,CD33+,cMPO+,CD19(Dim)+,CD56(Dim)+,CD64(Dim)
23	PB	Male	33	Relapse	M3	7.43	61	CD117+,CD13+,CD33+,CD64+,CD123+
24	PB	Female	46	New diagnosed	M3	30.21	65	CD117+,CD123+,CD13+,CD33+,CD64+
